# Nutritional Knowledge and Health Consciousness: Do They Affect Consumer Wine Choices? Evidence from a Survey in Italy

**DOI:** 10.3390/nu12010084

**Published:** 2019-12-27

**Authors:** Claudia Bazzani, Roberta Capitello, Elena Claire Ricci, Riccardo Scarpa, Diego Begalli

**Affiliations:** Department of Business Administration, University of Verona, 37129 Verona, Italy; claudia.bazzani@univr.it (C.B.); elenaclaire.ricci@univr.it (E.C.R.); riccardo.scarpa@univr.it (R.S.); diego.begalli@univr.it (D.B.)

**Keywords:** food choices, health consciousness, red wine, choice experiment, clean labels, alcohol content, nutritional knowledge

## Abstract

Wine is one of the few food products not subject to mandatory nutritional labelling, except for alcohol content. As such, health-related characteristics might be inferred by attributes related to production methods and alcohol content. This research focuses on the set of information currently reported on wine bottle labels, investigates the consumer’s use of such labels, and their preferences for information associated with ’naturalness’ such as clean labels and alcohol content. We conducted a survey on Italian consumers of red wine, which included a choice experiment. Results showed that health consciousness is an important driver in the use of wine labels. Estimates from a latent class model suggest that health consciousness, along with age, plays a significant role in defining consumer preference segments: the majority of our sample tended to prefer red wine characterized by ‘clean labels’, but younger and more health-conscious consumers showed a significant disutility for higher alcohol content. More traditional consumers revealed disutility for more unconventional ‘clean labels’, which were instead appreciated by a third group of consumers, called here ‘new clean trend lovers’. Preference for nutritional information such as lower alcohol content and clean labels distinguished the more health-conscious consumers, who belonged to the most likely preference class. Together, the results may suggest that nutritional information currently not mandatory for wine would be appreciated by a significant share of wine consumers.

## 1. Introduction

Consumer preferences for food products are evolving. An increasingly wider set of attributes is being considered by consumers when making food choices [1,2,3,4]. In the last few decades, more relevance has been given to sustainability attributes linked to, for example, nutritional, health-related properties, and environmental aspects of production processes [5]. Eating habits are indeed important from a societal point of view as they may have relevant consequences on citizen welfare and health. On the health side, the incidence of food-related illnesses and non-communicable chronic diseases such as, for example, obesity, diabetes, and cardiovascular diseases, has been increasing and is becoming a major problem for developed and developing countries [6]. This has increased medical spending and has also gradually amplified public awareness [7]. Thus, the nutritional aspects of food products and their relation to the individual’s future health have become particularly important for both policy makers and consumers [8].

In the context of wine, choices are particularly difficult because of the large number of different intrinsic and extrinsic product attributes [9]. Indeed, wine supply has been undergoing a strong process of differentiation, not only based on sensorial characteristics related to grape varieties and production methods, origin, and brand [10], but also on sustainability issues [11,12,13]. Some wineries are focusing on environmental impacts of wine production such as the type of agricultural practices (including, but not limited to, the use of energy-intensive pesticides and fertilizers), water and carbon footprints, the impacts on biodiversity, and sustainable waste management [14,15,16,17,18,19,20]. Indeed, following the environmental impacts of wine production, in the last decades, several codes of practices have emerged in the wine industries of California, New Zealand, Australia, South Africa, and Chile [15,21]. Other firms have directed their differentiation efforts on country of origin and on traditional production methods or health-related issues [22]. Some brands have started to promote sensible drinking campaigns to signal their care for consumers and to align with health-related public policies [23]. However, a debate in the literature exists regarding the appropriateness of such actions and their related intent [23,24,25,26]. 

Even if there are still issues of understanding and comprehension of the information contained in labels [27,28], labelling may constitute an important instrument to help fill the information gap between consumers and producers [29]. In particular, in relation to the health dimension of sustainability, the nutritional information in labels is viewed as one of the most effective ways to promote healthier food choices among consumers [30,31,32]. In the last 10–15 years, some of the issues related to nutritional properties and the health-effects of food products have been addressed by regulation. Wine remains one of the few food products not subject to regulation on nutritional labelling (for Europe see Regulation (EU) 1169/2011) [33,34]), even though discussion on how to change this is ongoing [35,36]. Indeed, the frequency and amount of alcohol consumption are strongly related to health. Although some benefits have been found for moderate wine consumption (especially for red) [37,38,39], in several western countries, wine constitutes a large share of the energy intake of individuals [40,41,42] and this may be associated with weight gain and non-communicable diseases [43,44]. More specifically, flavonoids, phenolics, and other phytochemicals contained in wine have been found to have antioxidant properties that may have a positive health effect in relation to chronic diseases [45,46]. Indeed, in relation to coronary heart diseases, the so-called “French paradox” suggests that wine consumption (especially red wine) may prevent artery damage by cholesterol and other circulatory issues [47,48]. Other studies suggest that moderate wine consumption can, in some cases, reduce the risk of cancer [49,50]. Moderate wine drinking seems to also positively affect other types of illnesses like bone density, ulcers, gall and kidney stones [50,51]. On the other hand, excessive wine consumption has been associated with an increase in health risks and mortality, in relation, for example, to alcoholism and its associated risks (liver cirrhosis, injuries related to drunk driving, and social behavior) and some types of cancer [39,48,51]. Alcohol poses further risks if consumed during pregnancy [52,53]. All of these effects are associated with high social costs [54]. Moreover, alcohol is an energy intense drink with low density nutritional value. Thus, drinking wine increases the calorie intake, and has been associated with food-related chronic health conditions [40,55,56].

Even if currently there are no mandatory labelling requirements concerning ingredient list and nutritional information [57,58], the wine economics literature has turned its attention toward consumer interest in nutritional labelling and health warning messages [59,60,61]. These studies highlight the growth in consumer interest in such indications and the contemporary low levels of knowledge about the nutritional aspects of wine consumption [62,63]. It was also observed that attitudes toward these labels depend on several factors including wine involvement, health consciousness, and attitudes toward nutritional information [33,63]. This is indeed consistent with the food-related literature, which highlights how health conscious and health-oriented consumers as well as those with higher than average levels of nutritional knowledge tend to generally read food labels more [31,32,64,65].

Food-related studies have also investigated consumer interest for the so-called ‘clean labels’ [66]. As Asioli et al. [66] emphasized, there is no clear definition of what a ‘clean’ label is; however, this concept is often associated with ’natural’ labels [67] such as those indicating organic, sustainable, ‘free from’ artificial additives/ingredients, or traditionally produced with minimal processing. Moreover, some studies have shown that “natural” products tend to be perceived by consumers as a healthier choice [68].

Specifically, the literature on consumer wine choices has focused on `clean’ labels related to aspects such as organic, biodiversity friendly, and biodynamic wine production processes [69,70,71,72], harvesting techniques, and the absence of additives [73,74,75,76]. This literature confirms the link between naturalness and health [77,78]. 

However, to the best of our knowledge, the existing literature that has explored consumer trade-offs for wine attributes that can be directly or indirectly linked to health issues is quite limited. This study focuses on the information set currently reported on (some) wine bottle labels and investigates consumer preferences for such wine attributes to evaluate if these may provide cues to promote healthier wine choices (e.g., lower alcohol content, sustainable agricultural practices) and, if so, among which types of consumers. In particular, we focused on ‘clean’ labels identified by Asioli et al. [36] as ‘organic’ and ‘natural’ labels (i.e., we did not focus on ‘free from artificial additives/ingredients’ labels) and labelled information directly linked to nutritional aspects such as alcohol content. We also aimed to explore whether attitudinal (e.g., health consciousness, wine interest, nutritional knowledge) and socio-demographic variables influenced consumer preferences for wine labels potentially linked to health [79].

Specifically, our research is directed at shedding light on three main questions:

- Is health orientation a driver of the consumer use of wine labels as in the case of food labels?
- What kind of labelled information (e.g., clean labels and/or alcohol content) directs wine choices toward more health-oriented consumers?
- What profiles characterize consumers interested in clean labels on wine and/or alcohol content?

Finally, we focused on at-home consumption as we aimed to investigate wine choices and eating habits. From a public policy point of view, this type of every-day behavior is interesting for studying and promoting healthier and more sustainable eating. Indeed, food consumption patterns and preferences for different types of product attributes may also differ depending on the eating occasion, the choice context, and the purchasing occasion [80,81,82,83], and some literature has highlighted that being in non-designated eating places, or more generally, eating-out, are associated with less healthy choices [84].

## 2. Materials and Methods

To achieve the objectives of this study, we conducted an online survey on a sample of Italian red wine consumers. Respondents were recruited in Italy by an online panel provider, were aged 18 years or older (i.e., the legal drinking age in Italy), and self-declared consuming red wine at home more than once a month. The questionnaire was administrated online in November 2018. The sample was stratified by gender, age, and region of residence, and consisted of 278 valid respondents.

The survey was primarily devoted to the collection of information, on one side, on consumer attitudes toward wine and health-related aspects; on the other, on consumer preferences for red wine consumed at home. Consumer preferences were elicited through a choice experiment (CE), the design of which is described in Section 2.1.

Consumer attitudes were surveyed through a set of questions making use of previously validated scales, which were aimed at evaluating consumer behavior and attitudes. These included the following:

- health consciousness [85,86];
- use of food labels and use of wine labels [87];
- perception of own weight status [88];
- food nutritional knowledge (adapted from Drichoutis et al. [32]);
- wine interest (adapted from Bruwer and Huang [89]); and
- wine consumption consciousness [90].

Appendix A presents the list of items included in the questionnaire according to the chosen scales. A seven-point Likert scale was used to measure the respondents’ levels of agreement on statements.

Furthermore, the questionnaire investigated wine consumption habits (e.g., frequency, occasions, preferred types of wine) and wine purchasing behavior (e.g., purchasing factors, preferred wine shops) [9,91,92,93]. We also evaluated if the respondent was aware that the number of calories in wine was proportional to the alcohol percentage in volume. 

The final part of the questionnaire included questions about the respondents’ socio-demographic characteristics.

### 2.1. Choice Experiment Design

CEs are one of the most popular stated preference methods used in food marketing to elicit individuals’ preferences and willingness-to-pay (WTP) for a good or a service. Their popularity is due to their ability to simultaneously estimate the utility of different attributes and attribute levels. Moreover, CEs are consistent with well-established theories, namely Lancaster's economic theory of consumer behavior [94], which assumes that the total utility of a good can be segregated in partial utilities given by the different attributes of the good, and with random utility theory, which assumes that individuals make choices to maximize their utility under a budget constraint [95]. 

In this study, the product item was red wine in a 750 mL bottle. We selected a red wine because the latter presents a wider range of alcohol content than white wine. Moreover, red wine is believed to have a more positive impact on health than other types of wine [51,96].

To avoid any kind of bias derived from the provenance and origin of the brands, we referred to a red wine produced in Italy without any protected designation of origin or geographical indication. No additional information on brand, grape variety, or sensory characteristic was indicated on the label.

To achieve the study objective, the attributes and levels were selected in order to propose several types of red wine characterized by product cues and production practices potentially affecting the healthiness and naturalness of wine. Here, the intent was to focus the design on some of the most relevant cues considered by the contemporary wine consumer and used as new product differentiation options by wineries [97]. 

Two pilot tests on a sample of 81 (pilot 1) and 80 (pilot 2) respondents, respectively, were performed to test the CE and refine the implemented attributes and their levels.

Table 1 presents the selected attributes and their levels as used in the CE. Specifically, five attributes were used:

- sustainability certification;
- hand-picked grapes;
- unfiltered wine;
- alcohol content; and
- price.

The attribute related to ‘sustainability certification’ was described by three levels: (1) organic certification, represented through the ‘European Leaf’ logo; (2) biodynamic certification, using the logo of the private protocol ‘Demeter®’; and (3) a certification aimed at communicating the adoption of agricultural practices that protect or enhance biodiversity (i.e., the private protocol ‘Biodiversity Friend®’, supported by the WBA World Biodiversity Association). We used these product information features in our analysis, since attributes related to sustainable agricultural practices might be linked to healthier food [66,98]. To illustrate, organic, biodynamic, and environmentally friendly products are often perceived as healthier and more natural [97,98,99,100,101,102]. The literature suggests that an important motivation for purchasing organic products is related to health considerations [103,104,105,106] and that health-conscious consumers are more in favor of organic food [107]. Indeed, organic buyers are found to also care highly for healthy diets [103,105]. 

We considered two further claims, ‘hand-picked grapes’ and ‘unfiltered wine’, which we indicated, when present, graphically through a logo that we conceived anew for the purpose of this study. Hand harvested and unfiltered claims where selected given that they tend to recall traditional production methods and indicate low levels of automation and processing, which as Asioli et al. [66] explained, can be considered as aspects of ‘clean labelling’. Indeed, some of the literature has highlighted how food processing and ‘machine-made’ (vs. hand-made) processes tend to reduce the level of ‘naturalness’ perceived by consumers [108,109,110], and how natural products tend to be perceived as healthier by consumers [68]. 

Furthermore, we investigated the role of alcohol content in wine choices. This indication is particularly interesting as it is already available on all wine bottles and it is linked to calorie and energy content [111]. Four levels of alcohol by volume (ABV) were used: 11%, 12%, 13%, and 14%, which allowed us to include a widespread alcohol content percentage of red wine in the Italian wine market.

The price attribute had six levels (€2.10, €3.60, €5.10, €8.10, €11.10, and €14.10) to take into account the extended price differentiation characterizing the wine on offer in Italy ‘off-premise’ stores (e.g., supermarkets, wine shops, or by the producers) [112].

Based on Thiene, Franceschinis, and Scarpa [88], the allocation of attributes and attribute levels to product alternatives was designed by employing a WTP_b_ efficient design that used as Bayes priors the WTP values obtained from the estimation of a multinomial logit model (MNL) on data from the second pilot (Choice Metrics, Ngene v1.0.1, 2011). The final design consisted of sixty choice tasks divided in five blocks of twelve choice tasks each. Thus, each respondent was asked to face twelve choice tasks. Each choice task presented three alternatives: two different bottles and a ‘neither of these’ alternative in order to let respondents opt out of purchase in case they did not prefer either bottle on offer. The two bottles in the choice task were described by their respective back label. Prices were indicated under the pictures displaying the back labels. The order of appearance of the choice tasks was also randomized as the order of the two product alternatives for each choice task.

To provide a homogenous context, respondents were asked to imagine buying a 750 mL bottle of red wine for a home consumption occasion in their usual shop. For each of the 12 choice tasks, respondents were asked to indicate the wine they preferred or to opt out. To limit the hypothetical bias, the choice experiment was introduced by a ‘cheap talk’ script [113]. Specifically, respondents were warned about the potential problem of hypothetical bias and were asked not to overstate their true WTP and focus on their responses as if they were in a real-life setting [114].

### 2.2. Models Specification

#### 2.2.1. Ordinary Least Square (OLS) regression

In order to answer our first research question (i.e., whether health-oriented individuals tend to be more prone to use wine labels), we performed an Ordinary Least Square regression specified as follows:

(1) $$y_{n}\;=\;\alpha_{0}+\overset{K}{\underset{k=1}{\sum }}\alpha_{k}s_{kn}+\epsilon_{n}$$

where $y_{n}$ is the dependent variable; $\alpha_{0}$, is the intercept of the model; $s_{kn}$ corresponds to the *k*th explanatory variable of the model (*k* = 1 to *K*); $\alpha_{k}$ is the average increment in $y_{n}$ associated with a unitary variation of $s_{kn}$; $\epsilon_{n}$ is the independent and identically distributed (i.i.d.) random error with zero mean. In our case, we specified the dependent variable ($y_{n}$) as the mean value of the five items capturing the use of wine labels, while we included the following as explanatory variables ($s_{kn}$): (1) health consciousness; (2) attitudinal information, which in the literature regarding food choices, have been determined as drivers in food label use (nutritional knowledge and being on diet or not); (3) information related to the respondents’ interest/knowledge of the wine product (wine interest, wine consumption consciousness, knowledge of the relationship between energy value, and alcohol degree); and (4) socio-demographic information. 

Finally, as a robustness check, we also tested a similar model on food label use in general to test if these behavioral variables affected wine label use in a similar way to food label use.

#### 2.2.2. Discrete Choice Models

Regarding our research questions related to consumer preferences for a set of labels that could potentially be related to health issues, we used discrete choice models (DCMs) to estimate the utility function determining the CE responses.

In DCMs, the utility for individual *n* of choosing alternative *i* in the choice situation *t* can be specified as follows:

(2) $$U_{nit}\;=\;{\beta_{n}}^{\prime }x_{nit}\;+\varepsilon_{nit}$$

where $x_{nit}$ is the vector of the observed variables relating to alternative *i* and individual *n* in choice task *t*; $\beta_{n}$ is a vector of individual structural taste parameters characterizing choices; $\varepsilon_{nit}$ is the unobserved utility term, assumed to be an independently and identically distributed Gumbel (Extreme Value Type I).

In an MNL model, the probability that consumer *n* chooses alternative *i* in choice task *t* is defined as follows:

(3) $${\mathrm{Pr}}_{nit}\;\;\frac{\exp\left(\beta_{n}^{\prime }x_{nit}\right)}{\sum_{j=1}^{J}\exp\left(\beta_{n}^{\prime }x_{njt}\right)}$$

In this specification, it is assumed that the consumers’ preferences are heterogeneous as described in studies for food or wine products [115]. However, studies have shown that consumers have preferences that may cluster into classes. This heterogeneity specification allows for a more parsimonious model than other random parameter logit (RPL) models accounting for preference heterogeneity [116]. RPL models assume heterogeneity to occur with a continuous distribution, meaning that each individual is assumed to have his/her own preferences. However, heterogeneity can be described by a discrete mixture, namely, different groups of consumers might have different preferences across groups, but preferences are identical within the group [117]. Given our interest in defining different preference clusters of consumers, in this study, we chose to adopt a latent class model (LCM) which takes into account that, within each segment, consumer preferences are homogeneous, but preferences vary between these segments [116,118].

In LCMs, respondents can be probabilistically assigned to a finite set of C classes on the basis of their observed pattern of choices. Each class is characterized by a unique class-specific vector of utility parameters *β_c_* for each of the attributes in the choice task. Given membership to a class *c*, the probability of the sequence of choices *y_n_* over the *T* choice occasions can be described as:

(4) $${\mathrm{Pr}}_{n}(y|c,x_{nit})\;=\;\prod_{t=1}^{T}\frac{\exp\left(\beta_{c}^{\prime }x_{nit}\right)}{\sum_{j=1}^{J}\exp\left(\beta_{c}^{\prime }x_{njt}\right)}\;$$

Membership probabilities for each latent class *c* are also defined according to a logit probability as:

(5) $$\pi_{c}\;\;\frac{\exp\left(\;a_{c}+{\;\gamma }_{c}^{\prime }Z_{n}\right)}{\sum_{c=1}^{C}\exp\left(\;a_{c}+\;\gamma_{c}^{\prime }\;Z_{n}\right)}$$

where $Z_{n}$ is the vector of co-variates characterizing respondent *n*, and *γ* is the vector of associated parameters to be estimated, while *a_c_* is a class-specific constant. In estimation, for identification purposes, only C−1 sets of coefficients can be identified. For one arbitrary class *c* the vector *γ_c_* and *a_c_* are both set to zero for identification purposes. In our analysis, we included as co-variates the same variables we used in the linear regression to explain wine/food label use. If these co-variates are significant, then we can use this information to explain segment membership.

In order to facilitate the inclusion of attitudinal and socio-demographic information in our model specifications (both in the OLS and the LCM), these variables were specified as follows:

- Age = dummy variable with value equal to 1 if the respondent has the median age or is older, 0 otherwise;
- Gender = dummy variable with value equal to 1 if the respondent was a woman, 0 otherwise;
- Education level = dummy variable with value equal to 1 if the respondent has the median education level or a higher one, 0 otherwise;
- Health consciousness = mean value of the health consciousness scale;
- Nutritional knowledge = mean value of the correct answers (if answer is correct = 1, 0 otherwise) to the questions related to food nutritional knowledge;
- Weight status control = dummy variable taking value of 1 for individuals who stated to be on a diet or to control their weight status, 0 otherwise;
- Knowledge calories/alcohol in wine = dummy variable taking value equal to 1 if the respondent answered correctly to the question related to wine nutritional knowledge, 0 otherwise
- Wine consumption consciousness = dummy with value equal to 1 if the respondent has a score equal to the median or higher, 0 otherwise;
- Wine interest = mean of the scores from the wine interest scale;
- Food labels use = mean of the scores from the use of food label scale; and
- Wine labels use = mean of the scores from the use of wine label scale.

## 3. Results

### 3.1. Sample Description, Attitudes, and Knowledge on Health, Food, and Wine Label Use and Wine Drinking

Table 2 illustrates the respondents’ socio-demographic characteristics. The proportion of women was slightly higher than that of men, and all age groups from 25–34 to 64–74 years were almost equally represented. The level of education was high (51.44% of respondents completed high school), while the level of stated household income was medium to low range.

Most respondents were interested in wine: they tended to like it and considered it as an important consumption good in their life; however, they were less involved in wine-related activities such as reading books or magazines on the subject or attending wine events (Table 3). They were mostly aware about the link between food and heart disease prevention while they are less knowledgeable about the nutritional compounds of food and wine. Most respondents paid attention to their weight and were quite interested in using labels when choosing food and wine products, and were conscious about the constant monitoring of their health status.

### 3.2. Ordinary Least Square Estimation

In relation to the first of our research questions, we first investigated the association between a set of variables related to health consciousness, nutritional knowledge, and socio-demographics with label use. With regard to food labels, our results confirm previous research findings that associate health consciousness and nutritional knowledge to higher food label use [32,64,65]. Specifically, results indicate that—ceteris paribus—more health-conscious respondents and those with a higher nutritional knowledge tended to have more favorable attitudes toward food label use (Table 4). Consumers who were conscious of wine consumption tended to use food labels more; however, knowledge about the link between alcohol consumption and calorie intake does not seem to have had an effect. Unlike what is usually found in the literature, the socio-demographic variables failed to play a significant role in our sample. Other variables such as the desire to control bodyweight, and other more wine-related variables were also found to be insignificant with food label use.

Table 4 also depicts the results of the same model applied to wine labels. With regard to wine label use, the results concerning health consciousness and wine consumption consciousness were confirmed. In this model, another variable more strictly related to wine becomes relevant. Indeed, as may be expected, wine interest seems to play a significant role on wine label reading. Moreover, age was statistically significant, indicating that older consumers tended to use wine labels more than younger consumers.

### 3.3. Latent Class Model

Different steps were developed in order to select the best number of classes for our data. At first, we used model fit statistics criteria, which led us to identify seven classes. However, as Ruto, Garrod, and Scarpa [119] explain, parsimony in the number of classes and the identification of sufficient explanatory classes are important criteria to take into account when selecting the optimal number of groups. Accordingly, in our study, we selected a Latent Class Model with three classes (Table 5). 

In our econometric analysis, all the ‘clean labels’ were specified as dummy variables, taking a value of 1 if the label was present on the bottle of wine, or 0 otherwise. On the other hand, alcohol content and price were treated as continuous variables describing the experimentally designed levels. As Table 5 shows, Class 3 was considered as the reference class for the determination of the effect of individual characteristics.

On the basis of the LCM model utility parameter estimates, the three classes can be described as follows:

Class 1. The health-conscious wine consumer: This class represents the highest share of respondents (63% of probability). These are the consumers who are the most prone to purchasing a bottle of wine for home consumption, given the negative and statistically significant coefficient of the no-buy option. Their probability of purchasing a bottle of wine significantly increases when one of the clean labels is present, with the exception of the biodynamic certification. What distinguishes this segment from the others is the negative and statistically significant parameter of the alcohol variable, indicating that an increase in alcohol degree in wine decreases the respondents’ utility from choosing a bottle of wine. Moreover, we observe that this was the only segment of consumers who have a strong significant preference for nearly all clean labels including the unfiltered wine. Finally, as expected, with an increase in price, individuals tend to have a higher disutility for buying the bottle. Belonging to this class is determined by an increase in health consciousness and by being of a younger age.

Class 2. The opponents: This is the segment with the lowest probability of membership (18%). Given the insignificant and positive value of the opt-out coefficient, this group of individuals generally do not tend to choose to buy a bottle of red wine for home consumption. Their probability of purchasing the bottle increases only with more ‘traditional-processing’ (i.e., hand-picked grapes) and more widespread organic certifications, while it decreases in the case of unconventional claims (e.g., biodiversity friendly or biodynamic wine). Alcohol content is not statistically significant, while we observe that an increase in price further decreases the utility of the opponents to purchase a bottle of wine. 

Class 3. The new clean trend lovers: These consumers are not generally interested in buying a bottle of wine, but they have a significant preference for clean labels, except for organic certification, which represents the most widespread clean label in the wine market. However, it is important to specify that the coefficient of the unfiltered claim is borderline significant (*p* = 0.103). Even in this case, we did not observe any significant effect of alcohol content on the consumers’ utility formation, while price has a negative effect.

## 4. Discussion

Our first research question was directed at investigating whether health-oriented consumers were more prone to reading wine labels. The OLS estimation results showed that attitude toward reading food and wine labels was mainly favored by some attitudinal antecedents of personal status consciousness (i.e., health and wine consumption). These findings are in line with several previous studies carried out in different countries, for example, Drichoutis et al. [32], Aschemann-Witzel et al. [5], and Cavaliere et al. [64]. 

With regard to nutritional knowledge, we observed that this information explained food label use as confirmed in the literature review by Drichoutis et al. [65], but not wine label use. Instead, the opposite occurred with the wine interest variable. Namely, the latter attitude significantly affected only wine labels use, as Annunziata et al. [33] and Vecchio et al. [63] also found. However, if we consider food nutritional knowledge as a proxy for involvement with food product, this result might indicate that interest in product characteristics is an important factor for explaining the use of labels, as, indeed, is suggested by the literature (see Cavaliere et al., [64] in the case of food products or Annunziata et al. [59] and Bruwer and Huang [89] in the case of wine). 

On the other hand, the significant effect of wine interest might be interpreted as the attitude of wine consumers to also gather product information from additional sources such as reading wine magazines or books, and participating in wine events [9,22,23,51,81,89]. Therefore, from a marketing prospective, these findings would suggest that the communication process toward consumers is quite challenging for wine companies and the label is only one component of such process, although it is of relevance during the purchasing choice, especially at off-premise points of sale. This is particularly important in relation to the age of consumers, as highlighted by our results and by other previous studies [60,70,82]. 

In conclusion, on the basis of our OLS regression, in relation to our research question of whether health-oriented consumers tend to use wine labels more, as in the case of food labels, our data suggest that this is indeed the case. In particular, our results suggest that wine and food label use is driven by similar attitudes.

As for the second and third research questions, ‘which kind of labelled information directs wine choices for health-oriented consumers’ and ‘who are the consumers interested in clean labels and/or alcohol content’, the latent class analysis suggests the existence of a large cluster of consumers (Class 1) who are driven by their health consciousness when choosing a wine. They prefer hand-made products (hand-picked grapes and unfiltered wine) and sustainable product certifications. This cluster may potentially represent an important market target, estimated around 60% of our sample that corresponds to the contemporary young consumer, who is particularly keen on quality assurance information. The above results are in line with previous work on quality attribute certifications [17,19] and on young consumers [38,70]. 

Importantly, what differentiates these health-conscious consumers from the other two clusters is the aversion toward a higher degree of alcohol content. This was actually expected since the literature shows that more health-oriented consumers tend to quite negatively perceive those wines with high alcohol content (see Yoo et al. [90]; Drichoutis et al. [32]; Martin-Moreno et al [35]; Casini et al. [93]). 

The cluster of the ‘opponents’ includes consumers who are not price-sensitive. They prefer organic certification and hand-picked grapes to other more recent claims. These findings suggest that this group of consumers tends to be driven by conventional product cues during their choice while health-related awareness does not seem to influence their preferences. Therefore, organic production and traditional practices potentially act as the principal preference drivers of choice for about one fifth of our respondents. This group could be an interesting potential market to be targeted and developed by organic producers. This aligns with previous findings from Schäufele and Hamm [12]; Schleenbecker and Hamm [106]; Fotopoulos et al [103]; de Magistris and Gracia [79]; and Castellini et al. [22].

The third class is characterized by consumers attracted to more innovative sustainability-related labels. For these consumers, price represents a constraint, but they demonstrate an interest in new label claims. They prefer hand-made to industrial wines (highlighted by the hand-picked grapes claim) and innovative ways to produce eco-friendly wine, like biodynamic and biodiversity friendly protocols [11,36,101]. What seems to emerge is an interest only for innovative sustainability-related labels. This is also the case among consumers who have a strong disutility for higher priced products, which is interesting given that a common finding in the literature is that richer people—who can afford pricier wines—have the strongest preferences for such attributes [12]. Our results suggest that this type of consumer potentially represents one fifth of the market target and are therefore of interest to producers proposing new types of natural or sustainable production schemes. Moreover, we did not observe health consciousness as a determinant of membership to this group. 

## 5. Conclusions

Given the interest of health-conscious consumers toward alcohol content information and wine label information in general, we believe that our results promote the use of nutritional information, even in the case of wine products. Nutritional information on wine may indeed add value to the nutritional aspects of wine in comparison with other types of alcoholic drinks. Nutritional information on wine labels could also represent a way of diffusing more awareness and of favoring the consumption of less-alcoholic wine, and therefore, a more responsible drinking behavior. Specifically, our findings suggest that the inclusion of nutritional information on wine labels could incentivize a more conscious use of the product in the home context. Indeed, within a daily (Mediterranean) diet, wine—as it also happens with extra virgin olive oil—can be considered as a ‘functional’ food, since a moderated quantity of the product is supposed to have positive effects for health [63,90]. This policy could be particularly oriented toward Italian mature adults who are more prone to using wine labels than younger consumers. Moreover, the latter are shifting their wine drinking patterns from regular consumption to occasional consumption during weekends, preferring out-of-home contexts, outside meals, and high alcohol beverages [51,60,70,81,82]. Thus, new tools should be developed to provide information in this context [120,121].

Before closing, it is important to point out that our results need to be interpreted with caution due to the size and geographical scope of the sample of respondents. Moreover, it is important to point out that the results may suffer from the hypothetical nature of the experiment. However, wine is a well-known food product with which Italian consumers are familiar in the context of making purchase choices. Thus, at least the novelty bias should be reduced [1,122]. However, some of the labelled indications might be new and unknown to some respondents.

Future research could explore attribute non-attendance (ANA) in order to corroborate our conjecture that health conscious individuals tend to give more attention to nutritional information such as alcohol over clean labels. Moreover, we believe that future research would advance the literature by testing the effect of providing nutritional information on consumer preference formation for different wine cues such as clean labels or information regarding brand, origin, and variety.

## Figures and Tables

**Table 1:** Attributes and attribute levels used in the choice experiment.

Attribute	Attribute Levels	Indication on The Back Label
Sustainability certification	Organic	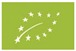
	Biodynamic	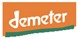
	Biodiversity friend®	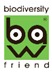
	None	-
Hand-picked grapes	-Present -Absent	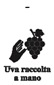
Unfiltered wine	-Present -Absent	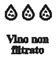
Alcohol content (% ABV)	11	11% vol
	12	12% vol
	13	13% vol
	14	14% vol
Price (€/750 mL bottle)	2.10	-
	3.60	-
	5.10	-
	8.10	-
	11.10	-
	14.10	-

**Table 2:** Socio-demographic characteristics of respondents (*n* = 278).

Variables	Sample
Age group (%)	
18–24 years	13.31
25–34 years	18.71
35–44 years	17.63
45–54 years	15.47
55–64 years	16.55
65–74 years	17.63
74+ years	0.72
Gender (%)	
Woman	52.52
Education (%)	
Comprehensive school	0.36
Intermediate school	12.23
High school	51.44
Bachelor degree	11.87
Master degree	18.35
PhD degree or post-doc	5.76
Total annual household net income (%)	
Less than €10,000	14.75
€10,000–€20,000	21.22
€20,001–€30,000	23.74
€30,001–€40,000	14.39
€40,001–€50,000	4.68
€50,001–€60,000	2.52
€60,001–€70,000	2.88
€70,001–€80,000	1.80
€80,001–€90,000	2.88
More than €90,000	2.52
I prefer not to answer this question.	8.63

**Table 3:** Respondents’ attitudes and knowledge on health, food, and wine label use and wine drinking (*n* = 278).

Variables	Sample
Weight status control (%)	
I’m trying to lose weight.	35.25
I’m trying to maintain my weight.	44.24
I don’t do anything to regulate my weight.	20.50
Wine consumption consciousness (mean)	
It is important to limit the amount of alcohol you consume	5.88
Wine Interest (mean)	
Wine is an important product to me.	5.38
I like wine.	5.86
I read magazines and books related to wine.	3.79
I take part in events which are related to wine (wine tourism, wine festivals, wine shows)	4.23
Food nutritional knowledge (%)	
Eating more fiber helps to prevent heart disease.	
Yes	71.58
No	10.43
Don’t know	17.99
Eating less salt helps to prevent heart disease.	
Yes	84.17
No	7.91
Don’t know	7.91
Eating more fruit and vegetables helps to prevent heart disease.	
Yes	83.81
No	8.27
Don’t know	7.91
Eating less saturated fats helps to prevent heart disease.	
Yes	73.74
No	7.19
Don’t know	19.06
Which has more calories: 100 grams of durum wheat pasta or 100 grams of white rice?	
Pasta	38.85
White rice	19.42
The same	13.31
Don’t know	28.42
Which fat do you think experts are saying is most important to cut down?	
Monosaturated fat	16.19
Saturated fat	55.40
Not sure	28.42
Knowledge calories/alcohol in wine (%)	
The amount of calories in wine is proportional to the alcohol percentage.	
True	48.56
False	20.86
Don’t know	30.58
Use of wine labels (mean)	
Reading wine labels takes more time that I can spend. (R)	4.50
Reading wine labels makes it easier to choose wines.	5.22
When I use wine labels, I make better wine choices.	5.10
Using wine labels to choose wines is better than just relying on my own knowledge about what is in them.	5.17
The information on wine labels is hard to interpret. (R)	4.56
Use of food labels (mean)	
Reading food labels takes more time that I can spend. (R)	4.12
Reading food labels makes it easier to choose foods.	5.38
When I use food labels, I make better food choices.	5.42
Using food labels to choose foods is better than just relying on my own knowledge about what is in them.	5.34
The nutritional information on food labels is hard to interpret. (R)	4.03
Health consciousness (mean)	
I reflect about my health a lot.	5.32
I am very self-conscious about my health.	5.40
I am alert to changes in my health.	5.46
I am usually aware of my health.	5.38
I take responsibility for the state of my health.	5.62
I am aware of the state of my health as I go through the day.	5.28

**Table 4:** Ordinary Least Square regression model results concerning reading food and wine labels.

	Food Label Use (Mean)	Wine Label Use (Mean)
Age	0.114 (0.104)	0.195 ** (0.097)
Gender	0.054 (0.101)	−0.111 (0.094)
Education level	−0.065 (0.108)	0.027 (0.101)
Health consciousness	0.364 *** (0.060)	0.247 *** (0.056)
Weight status control	0.126 (0.129)	−0.001(0.120)
Wine interest	−0.012 (0.046)	0.088 ** (0.043)
Knowledge calories/alcohol in wine	0.089 (0.104)	−0.036 (0.097)
Food nutritional knowledge	0.398 ** (0.191)	0.243 (0.179)
Wine consumption consciousness	0.223 ** (0.109)	0.212 ** (0.101)
Intercept	2.234 *** (0.330)	2.830 *** (0.309)
Model statistics		
R-square	0.240	0.175
No. of observations	278	278

***, ** denote significance at the 1%, 5%, and 10% level, respectively; 1 = Number in brackets are standard errors.

**Table 5:** Latent class model estimates.

	Parameters		
	Class 1	Class 2	Class 3
Attributes Variable	Coefficients	Coefficients	Coefficients
Hand-picked grapes	0.553 *** (0.076)	0.766 *** (0.168)	0.902 *** (0.322)
			
Unfiltered wine	0.201 *** (0.057)	−0.038 (0.147)	0.339 (0.208)
Organic	0.792 *** (0.085)	0.516 *** (0.170)	0.353 (0.308)
Biodynamic	0.130 (0.108)	−0.589 ** (0.233)	0.514 * (0.279)
Biodiversity	0.589 *** (0.087)	−0.542 *** (0.193)	0.830 *** (0.247)
Alcohol content	−0.095 *** (0.033)	−0.015 (0.078)	0.147 (0.102)
Price	−0.021 *** (0.006)	−0.050 ** (0.021)	−0.605 *** (0.041)
No-buy	−3.438 *** (0.423)	0.819 (0.950)	−1.264 (1.259)
Co-variates			
Constant	−1.203 (1.485)	−0.870 (1.735)	0
Weight status control	0.177 (0.483)	1.107 (0.751)	0
Wine interest	0.158 (0.186)	−0.136 (0.244)	0
Knowledge calories/alcohol in wine	−0.535 (0.386)	0.065 (0.520)	0
Food nutritional knowledge	−1.141 (0.826)	−1.047 (0.996)	0
Health consciousness	0.683 *** (0.264)	0.195 (0.335)	0
Wine consumption consciousness	−0.665 (0.442)	0.017 (0.584)	0
Education level	0.314 (0.465)	0.122 (0.595)	0
Age (dummy)	−1.674 *** (0.494)	−0.705 (0.605)	0
Gender	0.258 (0.422)	0.995 (0.607)	0
Size	63%	18%	19%
Model Statistics			
No. of observations	3336		
Log likelihood	−2695.389		
Akaike Information Criterion	5478.8		

***, **, * denote significance at the 1%, 5% and 10% level, respectively; 1 = Number in brackets are standard errors.

## Appendix A

**Table A1:** Scales used to assess consumer attitudes and knowledge on health, food, and wine label use and wine drinking used in this study.

Health consciousness [85,86] *(seven-point Likert from strongly disagree to strongly agree)* I reflect about my health a lot. I am very self-conscious about my health. I am alert to changes in my health. I am usually aware of my health. I take responsibility for the state of my health. I am aware of the state of my health as I go through the day. Use of wine labels (Adapted from Žeželj et al. [87]) *(seven-point Likert from strongly disagree to strongly agree)* Reading wine labels takes more time that I can spend. Reading wine labels makes it easier to choose wines. When I use wine labels, I make better wine choices. Using wine labels to choose wines is better than just relying on my own knowledge about what is in them. The information on wine labels is hard to interpret. Use of food labels [87] *(seven-point Likert from strongly disagree to strongly agree)* Reading food labels takes more time that I can spend. R Reading food labels makes it easier to choose foods. When I use food labels, I make better food choices. Using food labels to choose foods is better than just relying on my own knowledge about what is in them. The nutritional information on food labels is hard to interpret. R Weight status control (Adapted from Thiene et al. [88]) Which of the following statements best describe your actual status? - I’m trying to lose weight. - I’m trying to maintain my weight. - I don’t do anything to regulate my weight. Food Nutritional Knowledge [32] Which has more calories: 100 grams of durum wheat pasta or 100 grams of white rice? - Pasta - White rice - The same - Don’t know Which fat do you think experts are saying is most important to cut down? - Monosaturated fat - Saturated fat - Not sure Which fat do you think experts are saying is most important to cut down? - Monosaturated fat - Saturated fat - Not sure Do you think these behaviors help to prevent heart disease? *(Yes, No, don’t know)* - Eating more fiber - Eating more fiber - Eating less salt - Eating more fruit and vegetables - Eating less saturated fat Knowledge calories/alcohol in wine (adapted from Drichoutis et al.) [32] The amount of calories in wine is proportional to the alcohol percentage. - True - False - Don’t know Wine interest (adapted from Bruwer and Huang [89]) *(seven-point Likert from strongly disagree to strongly agree)* - Wine is an important product to me. - I like wine. - I read magazines related to wine. - I take part in events which are related to wine (wine tourism, wine festivals, wine shows). Wine consumption consciousness (mean) [90] *(seven-point Likert from strongly disagree to strongly agree)* - It is important to limit the amount of alcohol you consume
